# A comprehensive, genome-wide analysis of autophagy-related genes identified in tobacco suggests a central role of autophagy in plant response to various environmental cues

**DOI:** 10.1093/dnares/dsv012

**Published:** 2015-07-23

**Authors:** Xue-mei Zhou, Peng Zhao, Wei Wang, Jie Zou, Tian-he Cheng, Xiong-bo Peng, Meng-xiang Sun

**Affiliations:** 1 Department of Cell and Developmental Biology, College of Life Sciences, State Key Laboratory of Hybrid Rice, Wuhan University, Wuhan 430072, China; 2 Molecular Genetics Key Laboratory of China Tobacco, Guizhou Academy of Tobacco Science, Guiyang 550081, China

**Keywords:** autophagy, tobacco, gene expression, signalling, environmental stresses

## Abstract

Autophagy is an evolutionarily conserved mechanism in both animals and plants, which has been shown to be involved in various essential developmental processes in plants. *Nicotiana tabacum* is considered to be an ideal model plant and has been widely used for the study of the roles of autophagy in the processes of plant development and in the response to various stresses. However, only a few autophagy-related genes (ATGs) have been identified in tobacco up to now. Here, we identified 30 ATGs belonging to 16 different groups in tobacco through a genome-wide survey. Comprehensive expression profile analysis reveals an abroad expression pattern of these ATGs, which could be detected in all tissues tested under normal growth conditions. Our series tests further reveal that majority of ATGs are sensitive and responsive to different stresses including nutrient starvation, plant hormones, heavy metal and other abiotic stresses, suggesting a central role of autophagy, likely as an effector, in plant response to various environmental cues. This work offers a detailed survey of all ATGs in tobacco and also suggests manifold functions of autophagy in both normal plant growth and plant response to environmental stresses.

## 1. Introduction

Autophagy is an evolutionarily conserved mechanism for recycling of the cellular cytoplasmic contents or breaking down damaged materials in a cell, which plays essential roles in the remobilization of cytoplasmic components during nutrient starvation conditions.^1^ Identification of genes regulating autophagy in 1990s opened up the possibility to understand the molecular mechanism underlying autophagy and to explore the potential roles of autophagy in different physiological processes.^2^ In the past two decades, a series of autophagy-related genes (*ATGs*) required for autophagy have been characterized in yeast and mammals, and several important roles of autophagy in various developmental events, such as adaptation to starvation, regulation of metabolism, differentiation of cell types, clearance of damaged organelles, suppression of tumour, have been discovered.^2,3^ In addition, analyses of the networks of autophagy-related proteins revealed a canonical molecular pathway regulating the process of autophagy in yeast and mammals.^4^

Although remarkable progresses have been made in our understanding of the molecular mechanisms underlying autophagic pathways in yeast and mammals, molecular mechanisms and potential roles of autophagy in plants are still largely unknown. Recently, a great effort has been put on relevant studies and made it an energetic field. On the basis of sequence similarity to *ATGs* required for autophagy in yeast and mammals, a set of *ATGs* have been identified in *Arabidopsis thaliana*^5,6^ and *Oryza sativa*,^7^ respectively, and several roles of autophagy in plant development have been elucidated, e.g. leaf starch degeneration,^8^ tracheary element differentiation,^9^ hypertensive cell death,^10^ senescence,^11,12^ stress responses,^13^ life span extension,^14^ maintenance of peroxisomal quality,^15–17^ and anther development.^18^ In addition, core molecular machinery of autophagy was approved to be conserved among plants, yeast and mammals.^4,6^ However, the overall and specific roles of autophagy under normal and stress conditions, and their regulatory pathway underlying autophagy in plants, are still largely unknown.

*Nicotiana tabacum*, a traditional model plant, is assumed to originate from a hybridization event between ancestors of *Nicotiana sylvestris* and *Nicotiana tomentosiformis* ∼200,000 yrs ago.^19^ It is considered to be an ideal model plant for the study of autophagy in the processes of plant development and in the response to various severe environmental factors.^20–22^ However, only a few *ATGs* have been identified in tobacco. To facilitate our understanding of the roles of autophagy in plant development and molecular mechanism underlying it, it is essential to identify *ATGs* in tobacco. A systematic survey of *ATGs* in draft genomes of *N. tabacum* and expression sequence tags deposited in NCBI was performed, and comprehensive analyses of the expression patterns of *ATGs* under both normal and stress conditions were also carried out to gain insights into their putative roles in plant development under normal and in plant response to ill-suited environments.

## 2. Methods and materials

### 2.1. Plant materials

*Nicotiana tabacum* L. cv. Petite Havana SR1 plants were grown under 16 h/8 h light/dark cycles, at 25°C in greenhouse.

### 2.2. Identification *ATGs* in tobacco

To identify *ATGs* based on the draft genomes of *N. tabacum*,^23^ the program tBlastn using autophagy-related protein sequences in *A. thaliana* and *O. sativa* was performed in National Center for Biotechnology Information (NCBI). The DNA fragments and EST sequences related to *ATGs* were collected, respectively. Sequence assembling and open reading frame (ORF) analysis of each contig were performed using ContigExpress and OMEGA, respectively. After ORF analysis, BLASTP analysis with intact or partial deduced protein sequences of each contig was performed. The contigs corresponding to autophagy-related protein sequences based on the returning information were selected as candidates for further study.

### 2.3. Isolation of full-length cDNA of each *ATG* in tobacco

After ORF and BLASTP analyses of each contig, full-length cDNA of *ATG* candidates was obtained through an electronic cloning method or a rapid amplification of cDNA ends (RACE) approach. Total RNA isolated from anthers and leaves was used as a template to synthesize first-strand cDNA with the SMART RACE cDNA Amplification Kit (Clontech), and all reactions were performed according to the manufacturer's instructions. And then, full-length cDNA of each *ATG* was further confirmed through RT-PCR with specific primers at the 5′ and 3′ end, respectively.

### 2.4. Protein sequence and phylogenetic analysis

To analyse the relationships of autophagy-related genes identified in *N. tabacum* with that in *A. thaliana* and *O. sativa*, a multiple sequence alignment of ATG protein sequences was conducted with Clustal X ver. 1.81 program using the default multiple alignment parameters. The tree was constructed with MEGA 5.1 software using a maximum parsimony method.

### 2.5. RNA isolation and RT-PCR

Total RNA was extracted from leaf, root, stem, anther and pollen using TRI Reagent Solution (Ambion), and total RNA of seeds at different stages were extracted with RNAqueous™ (Ambion). All total RNA were treated with RNase-free DNase I (Promega) and cDNA were synthesized using PrimeScript Reverse Transcriptase (TaKaRa) under the condition recommended by the manufacturer procedure. Semi-quantitative RT-PCR was carried out in a 50-µl PCR mixture containing 5 µl of 10× Ex Taq buffer, 2.5 mM MgCl_2_, 200 µM dNTPs, 0.2 µM of primers, 1.2 U of Ex Taq DNA polymerase (Takara) and cDNA prepared from different tissues. Glyceraldehyde-3-phosphate dehydrogenase (GAPDH) was used as a control for normalization of cDNA prepared from different tissues. Detailed PCR conditions are described as follows: initial step for denaturation at 94°C for 2 min; then 35 cycles of denaturation at 94°C for 30 s, annealing at *T*_m_ −5°C for 30 s; extension at 72°C for 1 min and a final extension at 72°C for 5 min. Quantitative real-time reverse transcription PCR (RT-qPCR) was conducted for expression pattern analysis according to the previous procedure.^24^

### 2.6. Different stress treatments for tobacco seedlings

For different stress treatments, tobacco seeds were germinated in modified MS medium at 28°C for 2 weeks. Two-week-old seedlings were then transferred to different conditions for stress treatment. For carbon starvation, 2-week-old seedlings were transferred into modified MS medium without sucrose for 4, 16, 24 and 48 h, respectively. For nitrogen starvation, 2-week-old seedlings were transferred into nitrogen-free MS medium, in which NH_4_NO_3_ and KNO_3_ were replaced by KCl. For salt treatment, 2-week-old seedlings were transferred into modified MS medium containing 250 mM NaCl for 4 h. For cold treatment, 2-week-old seedlings were cultured in modified MS medium in 4°C for 4 h. For drought treatment, seedlings were kept in filter paper for 4 h at 28°C. For dark treatment, 2-week-old seedlings were kept in dark for 48 h at 28°C. For hormone treatment, 2-week-old seedlings were transferred into modified MS medium containing 1 µM naphthalene acetic acid (NAA), 5 µM 2,4-dichlorophenoxyacetic acid (2,4-D), 25 µM abscisic acid (ABA) or 5 µM gibberellic acid (GA_3_), 500 µM salicylic acid (SA) and 500 µM jasmonic acid (JA), for 24 h, respectively. For heavy metal treatment, 2-week-old seedlings were transferred into modified MS medium containing 40 µM CdCl_2_, 40 µM NiSO_4_, 40 µM ZnCl_2_, 20 µM CuSO_4_ or 100 µM MnCl_4_ for 24 h, respectively.

## 3. Results

### 3.1. Collection and identification of *ATGs* in tobacco

**Figure 1.**
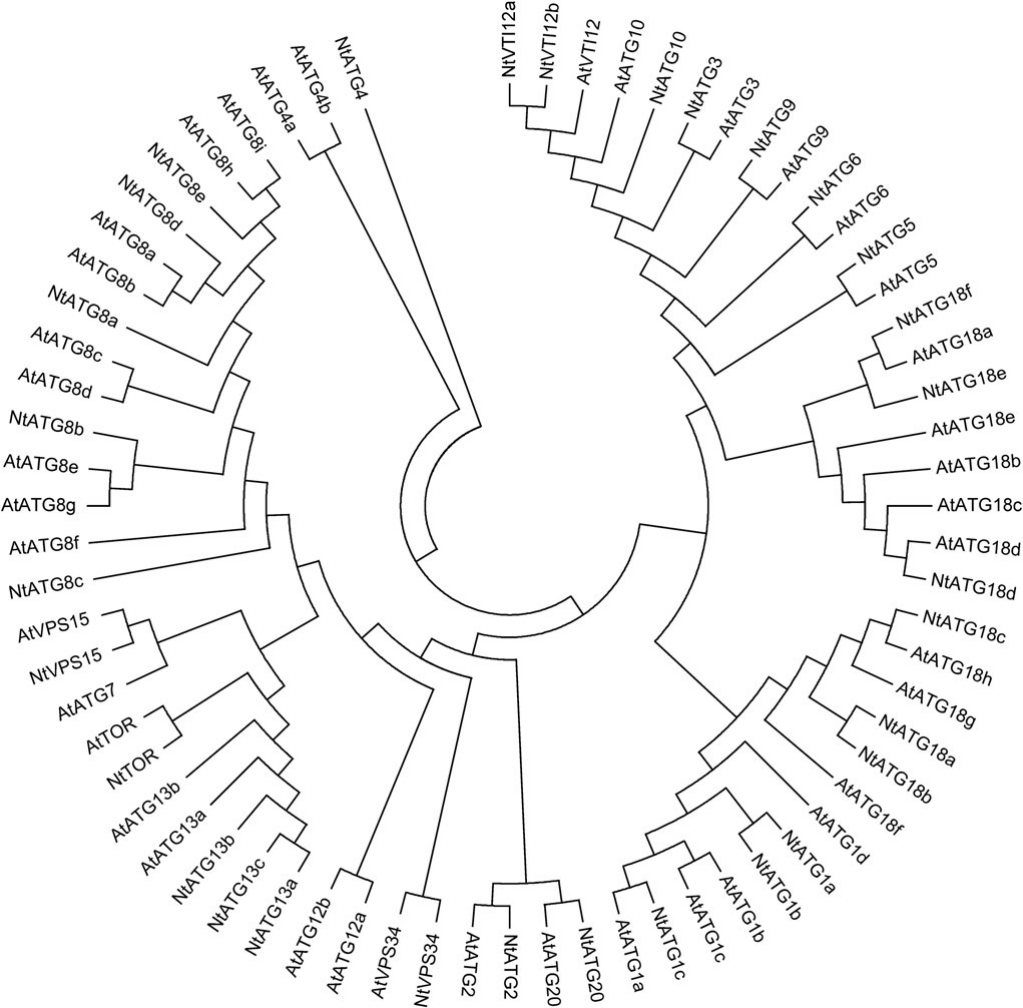
Phylogenetic relationship of ATGs from *N. tabacum* and *A. thaliana.* The tree was calculated with MEGA 5.1 software using the maximum parsimony method.

**Figure 2.**
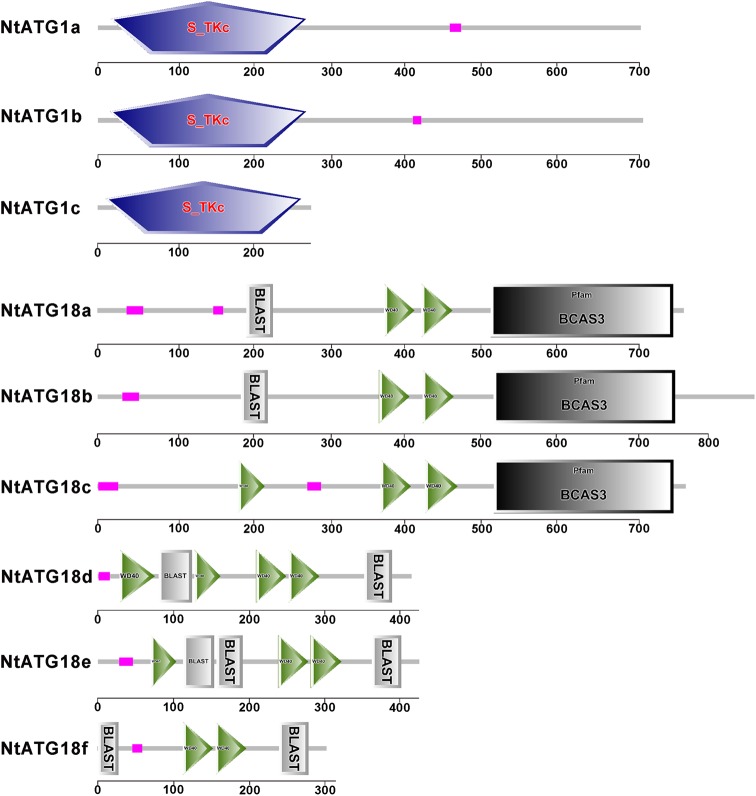
The structure divergence of ATG1s and ATG18s. This figure is available in black and white in print and in colour at *DNA Research* online.

To identify *ATGs* in tobacco, the tBlastn program using different autophagy-related protein sequences from *A. thaliana* and *O. sativa* was performed. Returned sequences related to *ATGs* were collected and assembled using ContigExpress, and redundant sequences were omitted manually. Then, a total of 30 individual contigs related to *ATGs* in tobacco were obtained. Full-length cDNA of them were obtained through an electronic cloning method or a RACE technique, and were further confirmed through RT-PCR with gene-specific primers. The detailed information of each gene was described in Table 1. To confirm that the 30 putative *ATG* homologues in tobacco are indeed *ATGs*, the deduced ATG protein sequences were analysed in the Pfam database and their sequence similarities to known ATG proteins in *A. thaliana* were analysed. The returned information of each ATG in the Pfam database were listed in Table 1, suggesting that all 30 *ATGs* in tobacco could be considered as true *ATGs*. Phylogenetically, each ATG protein sequence exhibits high similarities to their homologue in *A. thaliana* (Fig. 1 and Supplementary Fig. S1). However, several ATG groups (ATG1, ATG8, ATG10 and ATG18) in *A. thaliana* and *N. tabacum* were separated by ATGs in *O. sativa* in the phylogenetic tree (Supplementary Fig. S1), indicating the diversification of ATG evolution in different ATG groups. Generally, predicated 30 ATGs in tobacco could be divided into 12 different ATG groups and 4 relatives, and among them, the following groups comprise multiple isoforms: ATG1, ATG8, ATG13, ATG18 and VTI12. The composition of domains in each subgroup is similar with two exceptions (ATG1 and ATG18). ATG1c shows a similar serine/threonine-protein kinase domain at N-terminal to that of ATG1a and b, but lacks the C-terminal structure as shown in ATG1a and b (Fig. 2). A similar phenomenon was also observed in the ATG18 group. Two different subgroups could be divided according to the C-terminal structure. They show a similar N-terminal structure, but lacking of a C-terminal BCAS3 domain in ATG18d, ATG18e and ATG18f (Fig. 2). The composition of conserved domain in other groups is similar to that in *Arabidopsis*, indicating that the core machinery of autophagy is conserved in different angiosperms.

**Table 1.** The *ATG*s in tobacco

Group	Gene name	Accession no.	ORF (bp)	Predicated protein information				
				No. of amino acids	Mw (kDa)	PI	Signal peptide	Predicted function
ATG1	NtATG1a	KR336556	2,091	696	77.3	7.67	–	Serine/threonine-protein kinase
	NtATG1b	KR336557	2,073	690	76.7	6.13	–	Serine/threonine-protein kinase
	NtATG1c	KR336558	849	283	31.9	6.50	–	Serine/threonine-protein kinase
ATG2	NtATG2	KR336559	5,943	1,980	217.2	5.27	–	Autophagy-related protein 2
ATG3	NtATG3	KR336560	945	314	35.6	4.7	–	Autophagocytosis-associated protein 3
ATG4	NtATG4	KR336561	1,188	396	43.7	4.75	–	Peptidase family C54
ATG5	NtATG5	KR336562	1,116	371	41.3	5.06	–	Autophagy protein Apg5
ATG6	NtATG6	KR336563	1,674	557	5.57	62.8	–	Autophagy protein Apg6
ATG8	NtATG8a	KR336564	360	119	13.7	9.1	–	Autophagy protein Atg8
	NtATG8b	KR336565	369	122	14	6.61	–	Autophagy protein Atg8
	NtATG8c	KR336566	369	122	14	7.85	–	Autophagy protein Atg8
	NtATG8d	KR336567	372	123	14.1	6.61	–	Autophagy protein Atg8
	NtATG8e	KR336568	372	123	14.1	6.61	–	Autophagy protein Atg8
ATG9	NtATG9	KR336569	2,574	857	99	6.32	–	Autophagy protein Apg9
ATG10	NtATG10	KR336570	756	251	28.3	4.85	–	Autophagocytosis-associated protein
ATG13	NtATG13a	KR336571	1,827	608	67.4	8.62	–	Autophagy-related protein 13
	NtATG13b	KR336572	1,815	604	66.7	8.68	–	Autophagy-related protein 13
	NtATG13c	KR336573	1,827	608	67.5	8.62	–	Autophagy-related protein 13
ATG18	NtATG18a	KR336574	2,316	771	83.1	8.63	–	WD-40 repeat containing protein
	NtATG18b	KR336575	2,586	861	93.1	8.33	–	WD-40 repeat containing protein
	NtATG18c	KR336576	2,310	769	83.8	7.30	–	WD-40 repeat containing protein
	NtATG18d	KR336577	1,251	416	45.8	7.15	–	WD-40 repeat containing protein
	NtATG18e	KR336578	1,281	426	47.8	8.27	–	WD-40 repeat containing protein
	NtATG18f	KR336579	912	303	33.5	8.88	–	WD-40 repeat containing protein
ATG20	NtATG20	KR336580	1,206	401	46.1	7.68	–	Sorting nexin 1-like protein
VTI12	NtVTI12a	KR336581	666	221	25	9.18	–	Vesicle transport v-SNARE protein
	NtVTI12b	KR336582	666	221	24.8	8.47	–	Vesicle transport v-SNARE protein
VPS15	NtVPS15	KR336583	4,659	1,552	173.1	6.32	–	Serine/threonine-protein kinase
VPS34	NtVPS34	KR336584	2,445	814	93.2	6.33	–	Phosphoinositide 3-kinase
TOR	NtTOR	KR336585	7,782	2,593	291.8	6.90	–	Serine/threonine-protein kinase

**Figure 3.**
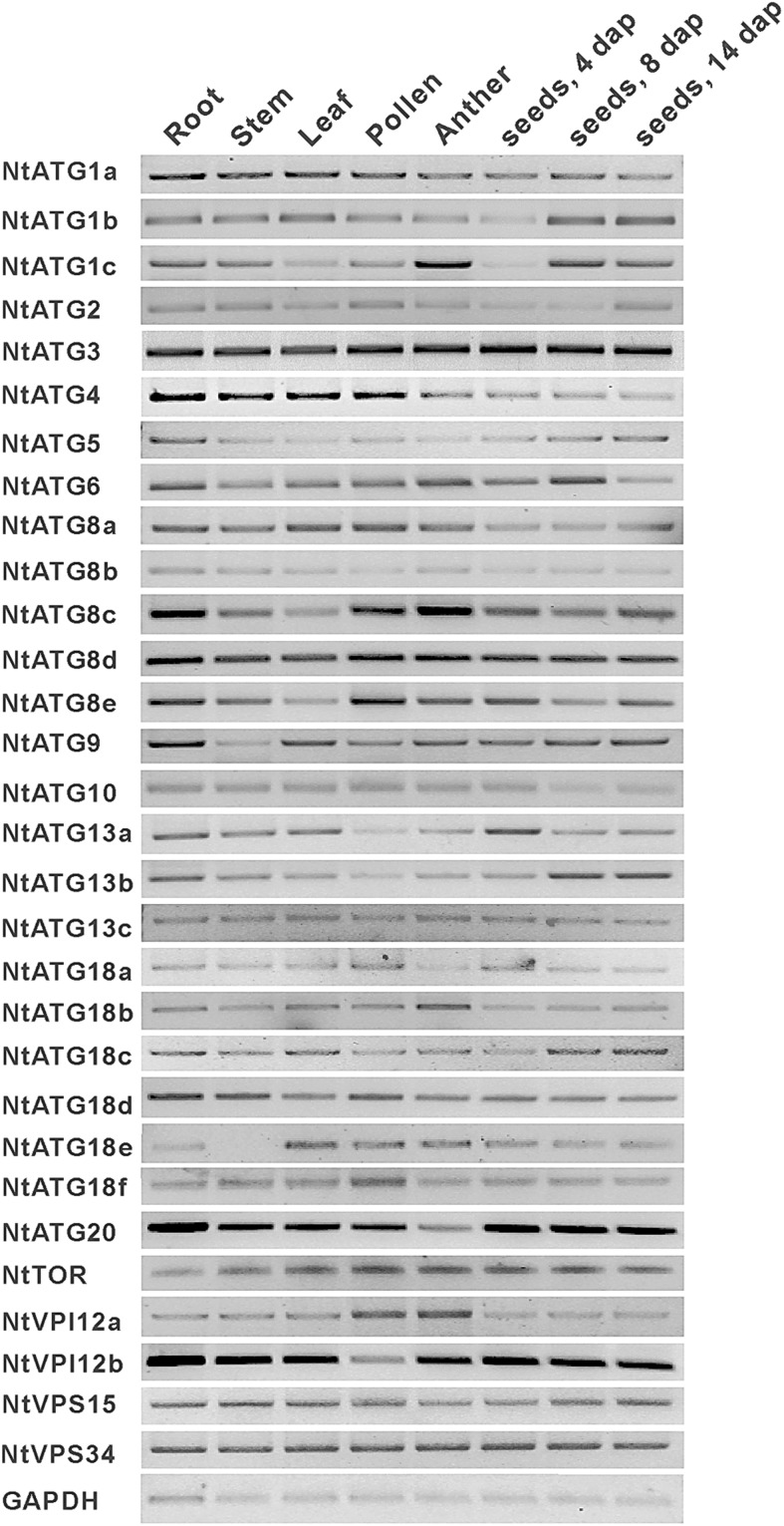
RT-PCR examination of the transcripts of *ATGs* in *N. tabacum.* The cDNA prepared from root, stem, leaf, pollen, anther, seeds (4, 8 and 14 days after pollination, respectively) were selected as templates for PCR. GAPDH was used as the control.

To further confirm the existence of predicated *ATGs* in tobacco, cDNA prepared from root, stem, leaf, pollen, anther and seed at different stages were selected as templates for RT-PCR. House-keeping gene *GAPDH* was used as the control for PCR. The transcripts of 30 predicated *ATGs* could be detected in different tissues of tobacco as shown in Fig. 3. Interestingly, all of them show a universal expression pattern in the eight tissues tested. These data suggest that all predicted *ATGs* are exactly existed in tobacco and autophagy may play a house-keeping role in the process of plant development.

### 3.2. Expression profile of *NtATGs* under normal growth environments

**Figure 4.**
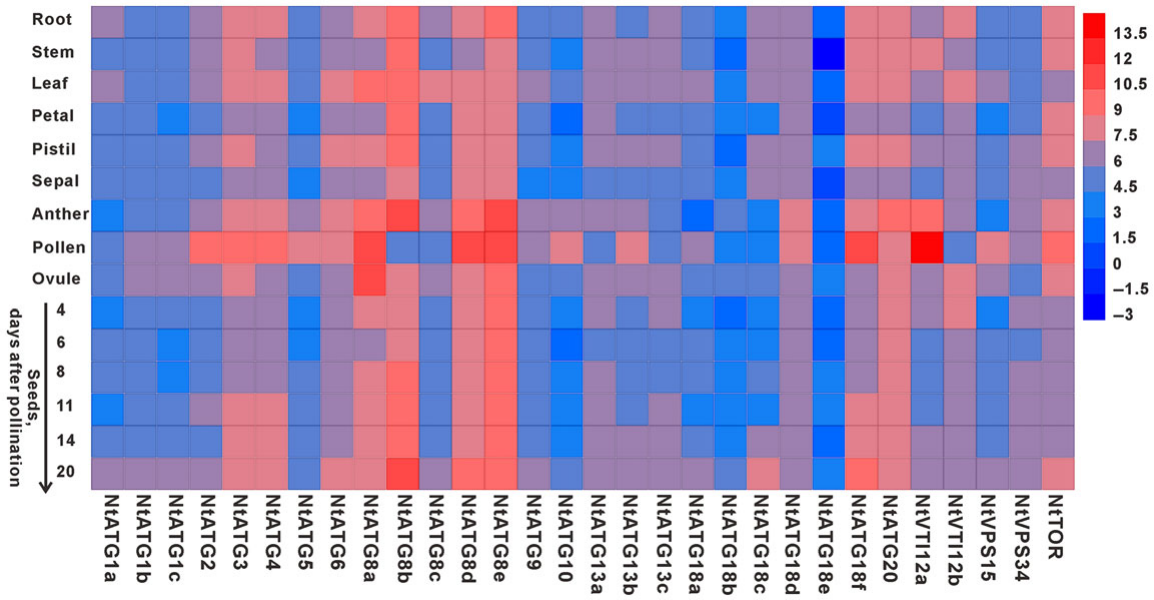
Expression profile of *NtATGs* in different tissues under normal conditions of plant growth. Expression profile of *NtATGs* in tobacco, which is constructed based on the relative expression level of each *ATGs* in different tissues. The expression level was normalized to GAPDH (AJ133422), Polyubiquitin (GQ281244), Actin (GQ281246) and Elongation factor 1a (AF120093). Blue box indicates the lower transcriptional level of *ATGs*, whereas red box indicates the higher expression level of *ATGs*. Scale bar represents fold change (log2 value).

There are growing evidences, suggesting that autophagy plays critical roles in the processes of plant development under normal conditions such as leaf starch degradation,^8^ root cell growth,^25^ rubisco degradation during leaf senescence,^26^ programmed cell death (PCD) of suspensor in somatic embryos of *Picea abies*,^27^ and maintenance of peroxisomal quality.^15–17^ To explore their potential roles in the processes of plant development, it is essential to study their expression pattern under normal growth conditions. RT-qPCR experiments were thus carried out, based on the cDNA from different tissues including leaf, stem, root, petal, sepal, anther, pollen, pistil, ovule and seeds at different developmental stages. The relative expression level of each *ATG* in different tissues was detected and compared with each other. Heat map analysis based on the relative expression level of each *ATG* was also performed, and an overview of the expression profile of *ATGs* in tobacco is presented in Fig. 4. Each *ATG* in tobacco exhibits a relatively broad expression pattern, which differ from the expression pattern of *ATGs* in *O. sativa.*^7^ Some tissue specifically expressed *ATGs*, such as *OsATG1b* and *OsATG8d*, have been identified.^7^ In contrast to that in *O. sativa*, the transcripts of each *ATG* in tobacco could be detected in all tissues tested, indicating a universal role of autophagy in the process of plant development. However, majority of *ATGs* show a relatively higher expression level in pollen/anther compared with that in other tissues tested. And, four homologues of ATG8 (*NtATG8a*, *NtATG8b*, *NtATG8d* and *NtATG8e*) show a relatively high expression level among all *ATGs* tested, which was thought to be required for autophagosome formation and reliable markers for the induction and progression of autophagy. Whereas four ATG18 group members (*NtATG18a*, *NtATG18b*, *NtATG18c* and *NtATG18e*) in ATG9 recycling complex show a relatively low expression level among all *ATGs* tested.

**Figure 5.**
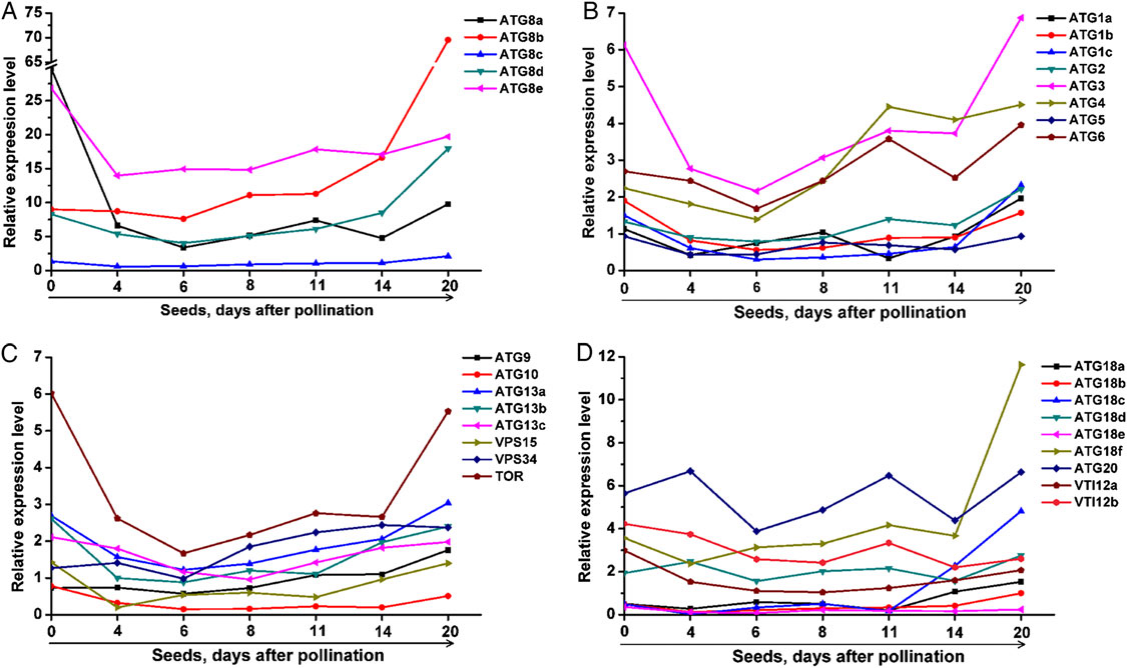
Dynamic changes in *NtATGs* during the process of seed development. Expression profile of *NtATGs* in tobacco, which is constructed based on the relative expression level of each *ATG* in seeds at different developmental stages. The expression level was normalized to GAPDH (AJ133422), Polyubiquitin (GQ281244), Actin (GQ281246) and Elongation factor 1a (AF120093). Data represent relative expression level (log2 value). This figure is available in black and white in print and in colour at *DNA Research* online.

Another striking characteristic of the expression profile is the temporal variations of *ATGs* during the process of seed formation. Three expression characteristics of *ATGs* could be observed in the whole process of seed development. The first is that the expression level of majority *ATGs* decreased upon fertilization with a few exceptions (*ATG8b*, *ATG9*, *ATG18d*, *ATG20* and *NtVPS34*). The second is that the expression levels of most *ATGs* are relatively stable during embryogenesis (seeds at 4–14 days after pollination). The third is that the expression peak of *ATGs* was found at the stage of seed maturation (Fig. 5). All these data suggest that autophagy may function at different developmental stages during the process of seed formation.

### 3.3. Expression pattern analysis of *NtATGs* under nitrogen and carbon starvation

**Figure 6.**
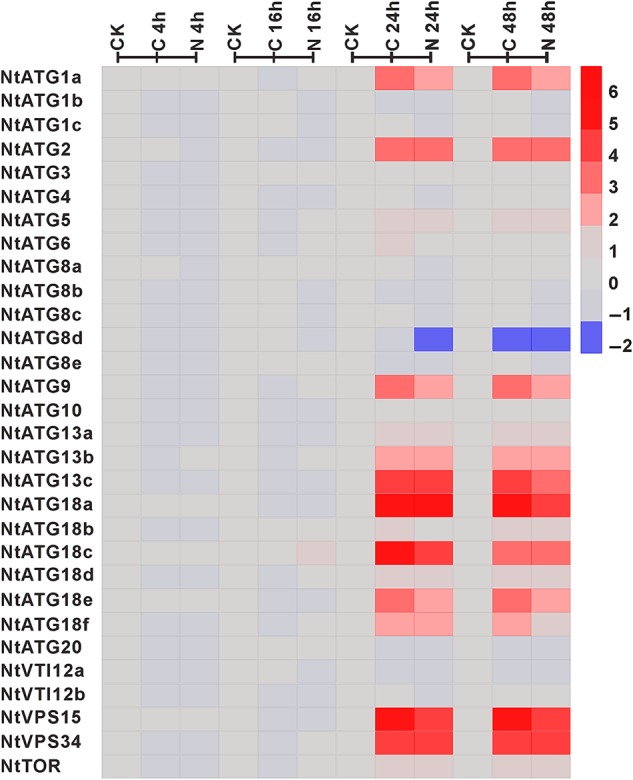
Expression profile of *NtATGs* in seedlings under sucrose and nitrogen starvation. Relative expression levels of each *ATG* in seedlings under sucrose and nitrogen starvation were normalized to GAPDH (AJ133422), Polyubiquitin (GQ281244), Actin (GQ281246) and Elongation factor 1a (AF120093). The expression level of each *ATG* was calculated and compared with that in seedlings under normal growth conditions. The expression level of each *ATG* in seedlings under normal growth conditions was indicated with grey box (0). Blue box indicates the lower transcriptional level of *ATGs*, whereas red box indicates the higher expression level of *ATGs*. Scale bar represents fold change (log2 value).

Autophagy is thought to function in protein breakdown and recycling of amino acids for survival in response to nutrient starvation, which is evolutionarily conserved from yeast to mammals. Some *ATGs* in plants have been approved to participate in regulating nutrient recycling under starvation conditions.^28^ To explore the potential roles of *NtATGs* during starvation conditions, their relative expression levels were investigated in seedlings that were treated in the condition of carbon and nitrogen starvation, respectively. To analyse the relative expression levels of *NtATGs* under carbon and nitrogen starvation, seedlings after treated for 4, 16, 24 and 48 h were collected, respectively. The expression levels of *NtATGs* in the plants were quantified and compared with the control to uncover the key *ATGs* involved in response to the starvation (Fig. 6 and Table 2). Our analysis showed that 17 *NtATGs* (*NtATG1a*, *NtATG2*, *NtATG5*, *NtATG6*, *NtATG9*, *NtATG13a*, *NtATG13b*, *NtATG13c*, *NtATG18a*, *NtATG18b*, *NtATG18c*, *NtATG18d*, *NtATG18e*, *NtATG18f*, *NtVPS15*, *NtVPS34* and *NtTOR*) were up-regulated (>2-fold expression change) and 1 *NtATG* (*NtATG8d*) was down-regulated (>2-fold expression change) in seedlings after carbon starvation 24 h. In addition, nine *NtATGs* (*NtATG1a*, *NtATG2*, *NtATG9*, *NtATG13c*, *NtATG18a*, *NtATG18c*, *NtATG18e*, *NtVPS15* and *NtVPS34*) show dramatically changed expression (>8-fold expression change).

**Table 2.** Overview of *ATGs* in tobacco response to different stresses

Name	Starvation		Hormone						Heavy metal					Other stress			
	–C	–N	NAA	2,4-D	ABA	GA_3_	SA	JA	Cu^2+^	Ni^2+^	Zn^2+^	Cd^2+^	Mn^2+^	Dark	Cold	Drought	Salt
NtATG1a	+++	+++	No	−	−	−−	+	++	+	+++	+++	+++	++	+++	++	+	+++
NtATG1b	No	No	No	No	No	No	No	No	No	No	No	No	No	No	−−−	−	−
NtATG1c	No	No	No	No	No	No	No	No	No	+++	No	No	No	+	−	No	No
NtATG2	+++	+++	++	++	++	No	No	+	+++	+++	+++	++	+++	+++	+	+	+++
NtATG3	No	No	No	No	No	No	No	No	No	No	No	No	No	No	−	No	No
NtATG4	No	No	No	No	No	No	No	No	No	No	No	No	No	No	−	No	No
NtATG5	+	+	+	+	No	−	No	No	No	+	+	No	+	−	No	No	+
NtATG6	+	No	No	No	No	No	No	No	No	No	No	No	No	No	−	No	No
NtATG8a	No	No	No	No	No	No	No	No	No	No	No	−	No	++	−−	No	No
NtATG8b	No	No	No	No	No	No	No	No	No	No	−	−	No	+	−	No	No
NtATG8c	No	No	No	No	No	No	No	No	No	No	No	No	No	No	+	−	No
NtATG8d	−	−	No	No	No	No	No	No	No	No	No	No	No	+	−	No	No
NtATG8e	No	No	No	No	No	No	No	No	No	No	No	No	No	No	−	No	No
NtATG9	+++	+++	++	++	++	No	No	+	+++	+++	+++	+++	+++	−	++	++	+++
NtATG10	No	No	No	No	No	No	No	No	No	No	No	No	No	No	−	−	−
NtATG13a	+	+	No	No	No	No	No	No	+	+	+	No	+	+	No	No	+
NtATG13b	++	++	No	No	No	−	No	+	+	+	++	+	+	++	No	No	+
NtATG13c	+++	+++	+++	++	++	No	No	+	+++	+++	+++	+++	+++	+++	+	++	+++
NtATG18a	+++	+++	No	No	No	−−	+	+++	+	+++	+++	+	++	+++	+	+	+++
NtATG18b	+	+	No	No	No	No	No	++	No	No	No	No	No	No	−	No	No
NtATG18c	+++	+++	No	No	No	−	++	+++	+	+	+++	+	+	+++	++	+	++
NtATG18d	+	+	No	No	No	−	No	No	+	+	+	No	+	−−−	−	No	No
NtATG18e	+++	+++	No	No	No	−−−	+	+	No	No	++	No	No	No	No	No	+
NtATG18f	++	++	+	No	No	No	No	No	+	+	++	+	+	+	No	No	++
NtATG20	No	No	No	No	No	No	No	No	No	No	No	No	No	+	−	No	No
NtVTI12a	No	No	No	No	No	No	−	No	No	No	No	No	No	++	−	−	−−−
NtVTI12b	No	No	No	No	No	No	No	No	No	No	No	No	No	No	No	No	No
NtVPS15	+++	+++	No	No	No	−−−	No	+	++	+++	+++	+++	+++	+++	No	No	+++
NtVPS34	+++	+++	++	++	++	−−	No	+	+++	+++	+++	+++	+++	+++	++	+	+++
NtTOR	+	+	No	No	No	No	No	No	+	+	+	+++	+	No	No	No	+

‘+’ and ‘−’ mean that the expression of *ATGs* were up-regulated and down-regulated by different stresses, respectively. The number of ‘+’ or ‘−’ means different fold change of relative expression levels. ‘+’ or ‘−’ means >2-fold change. ‘++’ or ‘−−’ means >4-fold change. ‘+++’ or ‘−−−’ means >8-fold change.

The duration of starvation treatment for the expression level changes is another focus in our study. The relative expression levels of all *NtATG*s in seedlings show no visible change after 4- and 16-h carbon starvation treatment. However, the relative expression levels of most *NtATG*s in seedlings increased dramatically after 24-h carbon starvation treatment and with a little further decrease in seedlings after 48-h treatment, indicating that the response of autophagy to starvation signals usually occurs after 16- to 24-h starvation treatment. More interestingly, all *NtATG*s show a similar response pattern under nitrogen starvation with that in carbon starvation treatment, indicating a common response mechanism to carbon and nitrogen starvation stresses.

### 3.4. Differential expression of *NtATGs* in response to stress treatments

**Figure 7.**
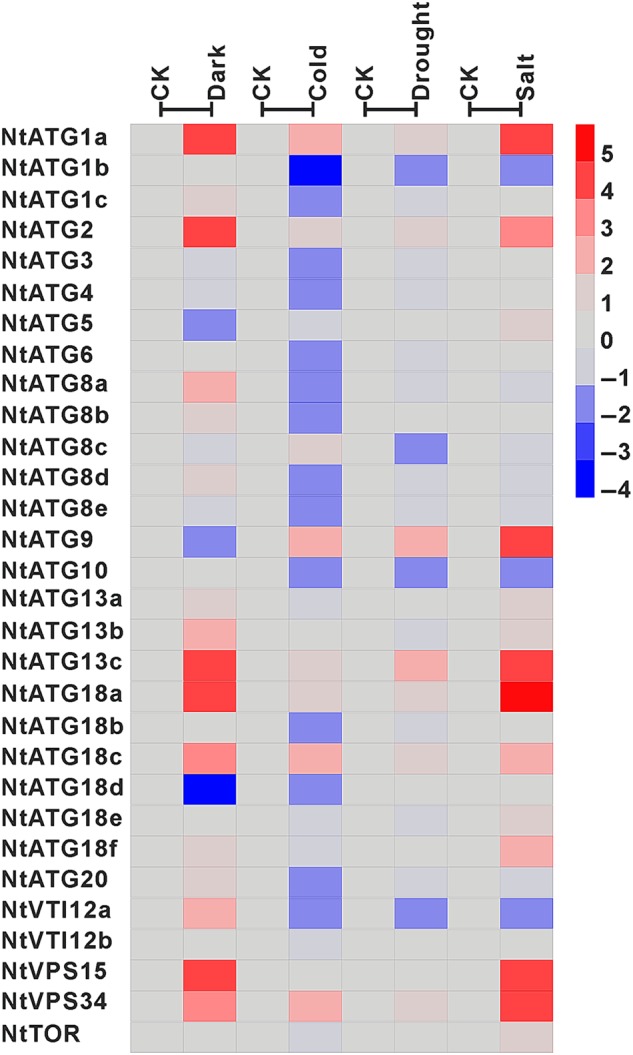
Expression profile of *NtATGs* in seedlings under dark, cold, drought and salt stresses. Relative expression levels of each *ATG* in seedlings under dark, cold, drought and salt stresses were normalized to GAPDH (AJ133422), Polyubiquitin (GQ281244), Actin (GQ281246) and Elongation factor 1a (AF120093). The expression level of each *ATG* was calculated and compared with that in seedlings under normal growth conditions. The expression level of each *ATG* in seedlings under normal growth conditions was indicated with grey box (0). Blue box indicates the lower transcriptional level of *ATGs*, whereas red box indicates the higher expression level of *ATGs*. Scale bar represents fold change (log2 value).

To gain insights into the potential roles of *NtATGs* in response to various stress treatments, their expression dynamics were investigated through RT-qPCR in tobacco seedlings subjected to dark, cold, drought and salt treatments. Generally, all of the *NtATGs*, except *NtVTI12b* in tobacco, are responsive to different stress treatments, and the relative expression levels of them changed significantly upon different stresses (Fig. 7 and Table 2). However, the response of *NtATGs* to different stresses varied significantly. Only six *ATGs* (*NtATG1a*, *NtATG2*, *NtATG13c*, *NtATG18a*, *NtATG18c* and *NtVPS34*) were commonly up-regulated by all the four different stress treatments, indicating a common role these *NtATGs* in dark, cold, drought and salt stresses. Whereas the expression of some *NtATGs* (*NtATG3*, *NtATG4*, *NtATG6*, *NtATG8e*, *NtATG18b*, *NtATG18e* and *NtTOR*) were responsive to some specific stress.

In response to dark treatment, the expression level of 19 *NtATGs* shows significantly changes. Most of them (*NtATG1a*, *NtATG1c*, *NtATG2*, *NtATG8a*, *NtATG8b*, *NtATG8d*, *NtATG13a*, *NtATG13b*, *NtATG13c*, *NtATG18a*, *NtATG18c*, *NtATG18f*, *NtATG20*, *NtVTI12a*, *NtVPS15* and *NtVPS34*) are up-regulated in seedlings under dark conditions (>2-fold expression change) and three *ATGs* (*NtATG5*, *NtATG9* and *NtATG18d*) were down-regulated (>2-fold expression change). Among them, only six *NtATGs* (*NtATG1c*, *NtATG8a*, *NtATG8b*, *NtATG8d*, *NtATG20* and *NtVTI12a*) and two *NtATGs* (*NtATG5* and *NtATG9*) were specifically up-regulated and down-regulated by dark treatment, respectively. Unlike dark treatment, a larger number of *NtATGs* (*NtATG1b*, *NtATG1c*, *NtATG3*, *NtATG4*, *NtATG6*, *NtATG8a*, *NtATG8b*, *NtATG8d*, *NtATG8e*, *NtATG10*, *NtATG18b*, *NtATG18d*, *NtATG20* and *NtVTI12a*) were down-regulated by cold stress (>2-fold expression change). For drought treatment, a relatively small number of *ATGs* (11 of 30 *ATGs*) show their response to drought stress (seven *ATGs* up-regulation and four *ATGs* down-regulation). For salt treatment, 14 *NtATGs* (*NtATG1a*, *NtATG2*, *NtATG5*, *NtATG9*, *NtATG13a*, *NtATG13b*, *NtATG13c*, *NtATG18a*, *NtATG18c*, *NtATG18e*, *NtATG18f*, *NtVPS15*, *NtVPS34* and *NtTOR*) and 3 *NtATGs* (*NtATG1b*, *NtATG10* and *NtVTI12a*) were up-regulated and down-regulated, respectively.

### 3.5. Differential expression of *NtATGs* in response to heavy metal stress

**Figure 8.**
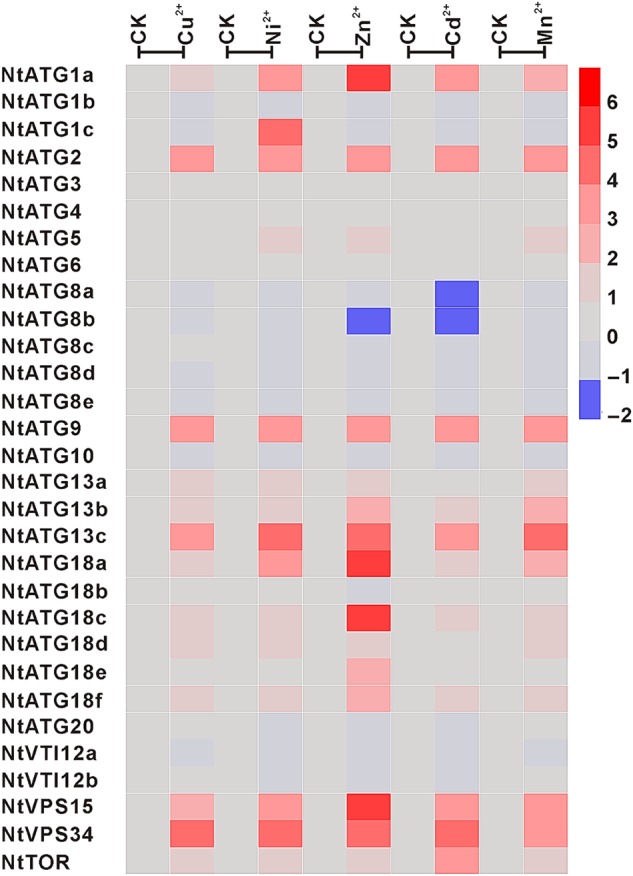
Expression profile of *NtATGs* in seedlings under heavy metal stresses. Relative expression levels of each *ATG* in seedlings under heavy metal stresses were normalized to GAPDH (AJ133422), Polyubiquitin (GQ281244), Actin (GQ281246) and Elongation factor 1a (AF120093). The expression level of each *ATG* was calculated and compared with that in seedlings under normal growth conditions. The expression level of each *ATG* in seedlings under normal growth conditions was indicated with grey box (0). Blue box indicates the lower transcriptional level of *ATGs*, whereas red box indicates the higher expression level of *ATGs*. Scale bar represents fold change (log2 value).

Heavy metals are major environmental pollutants, which are highly reactive and toxic to living cells. Correspondingly, organisms have adapted to multiple heavy metal stresses by developing various response mechanisms. In human haematopoietic stem cells, autophagy has been approved to be implicated in the response to Cd^2+^ stress.^29^ However, the role of autophagy in response to heavy metal stress in plants still remains unknown. To survey the putative roles of autophagy in plant response to heavy metal stress, it is a prerequisite to extensively analyse their expression dynamics in response to different heavy metal stresses. Thus, the relative expression levels of 30 *ATGs* were analysed in seedlings treated with five different heavy metals including Cu^2+^, Ni^2+^, Zn^2+^, Cd^2+^ and Mn^2+^ with a comparison to that in untreated seedlings (Fig. 8 and Table 2). Expression profile analysis data showed that the expression of 18 *ATGs* in seedlings are regulated by at least one heavy metal (>2-fold expression change), and the expression levels of other 12 *ATGs* (*NtATG1b*, *NtATG3*, *NtATG4*, *NtATG6*, *NtATG8c*, *NtATG8d*, *NtATG8e*, *NtATG10*, *NtATG18b*, *NtATG20*, *NtVTI12a* and *NtVTI12b*) show no visible changes. Among the 18 heavy metal-responsive *ATGs*, the expression levels of 11 *ATGs* are commonly up-regulated with 7 exceptions (*NtATG1c*, *NtATG5*, *NtATG8a*, *NtATG8b*, *NtATG13a*, *NtATG18d* and *NtATG8e*) in seedlings after treated with five different heavy metals, indicating a common mechanism of *ATGs* in the response to different heavy metal treatments. Interestingly, the expression of *ATGs* is more sensitive to Zn^2+^ treatment than to other heavy metals. However, how these heavy metals regulate autophagy remains to be elucidated in the further studies.

### 3.6. Differential expression of *NtATGs* in response to plant hormone treatment

**Figure 9.**
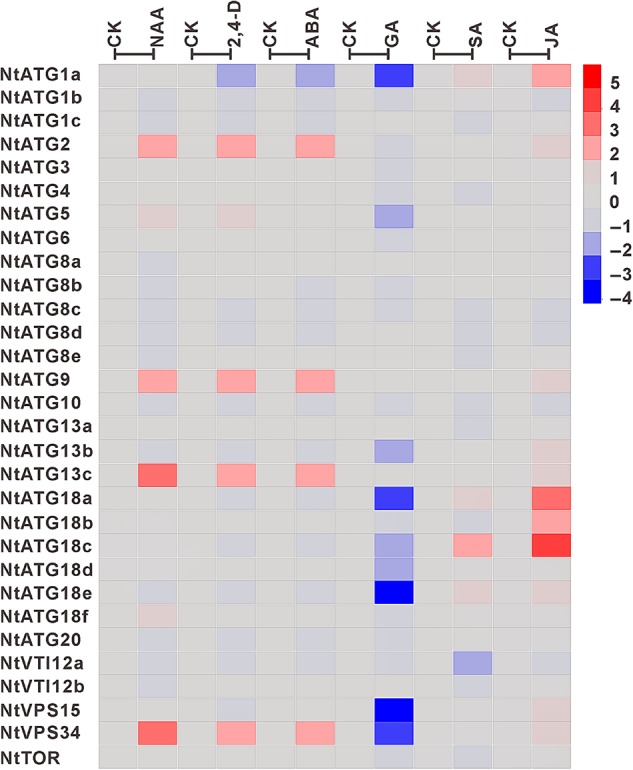
Expression profile of *NtATGs* in seedlings under different hormone treatments. Relative expression levels of each *ATG* in seedlings under different hormone treatments were normalized to GAPDH (AJ133422), Polyubiquitin (GQ281244), Actin (GQ281246) and Elongation factor 1a (AF120093). The expression level of each *ATG* was calculated and compared with that in seedlings under normal growth conditions. The expression level of each *ATG* in seedlings under normal growth conditions was indicated with grey box (0). Blue box indicates the lower transcriptional level of *ATGs*, whereas red box indicates the higher expression level of *ATGs*. Scale bar represents fold change (log2 value).

Plant hormones are a group of small signal molecules that exert powerful effects on a variety of developmental processes such as embryogenesis,^30^ root development,^31^ PCD^32^ and many other aspects of plant development by ‘regulating gene’ expression. In a recent report, SA signalling pathway was shown to be required for early senescence in *atg* mutant.^12^ However, the relationship between plant hormones and autophagy is still largely unknown. To test whether plant hormones could regulate the expression pattern of *ATGs*, the responses of *NtATGs* to different plant hormones were analysed in the present study. The relative expression levels of each *ATG* under six different hormone treatments including NAA, 2,4-D, ABA, GA_3_, SA and JA were investigated by RT-qPCR, and heat map analysis was conducted based on the relative expression level of each *ATG*. The data revealed that the expression levels of several *ATGs* in seedlings changed significantly when treated with different plant hormones. Notably, different ‘hormones’ have ‘diverse effects on the regulation of’ ATG ‘expression’ (Fig. 9 and Table 2)*.* After GA_3_ treatment, the ‘expression levels of nine’ *ATGs* (*NtATG1a*, *NtATG5*, *NtATG13b*, *NtATG18a*, *NtATG18c*, *NtATG18d*, *NtATG18e*, *NtVPS15* and *NtVPS34*) in seedlings were down-regulated significantly (>2-fold expression change). However, the expression levels of *ATGs* were usually up-regulated with a few exceptions in seedlings by other five hormones tested. After NAA treatment, the expression levels of six *ATGs* (*NtATG2*, *NtATG5*, *NtATG9*, *NtATG13c*, *NtATG18f* and *NtVPS34*) showed a significant increase (>2-fold expression change). The expression levels of five *ATGs* (*NtATG2*, *NtATG5*, *NtATG9*, *NtATG13c* and *NtVPS34*) and four *ATGs* (*NtATG2*, *NtATG9*, *NtATG13c* and *NtVPS34*) were increased significantly (>2-fold expression change) after 2, 4-D and ABA treatments, respectively. In addition, 4 *ATGs* (*NtATG1a*, *NtATG18a*, *NtATG18c* and *NtATG18e*) and 11 *ATGs* (*NtATG1a*, *NtATG2*, *NtATG9*, *NtATG13b*, *NtATG13c*, *NtATG18a*, *NtATG18b*, *NtATG18c*, *NtATG18e*, *NtVPS15* and *NtVPS34*) were up-regulated (>2-fold expression change) by SA and JA, respectively. That is to say, these five plant hormones (NAA, 2,4-D, ABA, SA and JA) usually exert opposite effects on the regulation of *ATG* expression compared with GA_3_, implying that they may have common mechanism on regulating autophagy occurrences in the process of plant development, but differ from that of GA_3_.

## 4. Discussion

Autophagy is an evolutionarily conserved cellular degradation process among yeast, mammals and plants.^4,6^ A set of genes involved in this process are called *ATGs* and has been extensively studied in yeast and mammals. More than 30 *ATGs* have been identified until now, and analyses of them unveiled multiple roles of autophagy and diverse aspects of molecular mechanism underlying it.^3^ On the basis of sequence similarity to autophagy-related proteins in yeast and mammals, 39 *ATGs* belonging to 20 different groups in *A. thaliana*^5,6^ and 33 *ATGs* belonging to 13 different groups in *O. sativa*^7^ were identified, respectively, suggesting the core components of autophagy are conserved among different species. In addition, these ATGs could be divided into four major functional groups including ATG1 kinase complex group, ATG9 recycling complex, phosphoinositide-3-kinase (PI3K) complex and ubiquitin-like conjugation system. However, the roles of them in plant development and in response to dynamic environment, as well as conservation of autophagy between monocotyledon and dicotyledon, are still largely unknown.

*Nicotiana tabacum* has been used as an ideal model plant for the study of autophagy in plants for many years.^20–22^ However, only a few *ATGs* have been identified up to now in tobacco. To facilitate our understanding of the molecular mechanism regulating autophagy, it is necessary to identify all the *ATGs* in tobacco genome. Thus, in the present study, 30 *ATGs* belonging to 16 different classes according to the sequence similarity to *ATGs* of *A. thaliana* have been identified. No homologues of *ATG7* and *ATG12* have been identified in the present study based on the draft genomic sequences of *N. tabacum* deposited in NCBI, and no EST sequences covering *ATG7* and *ATG12* were found according to available data. Most of the *ATGs* (*ATG2*, *ATG3*, *ATG4*, *ATG5*, *ATG6*, *ATG9*, *ATG10*, *ATG20*, *VPS15* and *VPS34*) comprise single member. Only a few of them (*ATG1*, *ATG8*, *ATG13*, *ATG18* and *VTI12*) contain multiple members, ranging from two to six in different groups (Table 1). However, majority of *ATGs* in *O. sativa* have multiple isoforms with four exceptions (*ATG5*, *ATG7*, *ATG12* and *ATG16*), indicating that plants may have species-specific mechanism regulating autophagy. In *N. tabacum*, the compositions of domain within each class are similar with two exceptions (ATG1 and ATG18), suggesting possible functional redundancy of different members in the same group. ATG1c shows a similar serine/threonine-protein kinase domain at N-terminal with 1a and 1b, but lacks a similar C-terminal structure that in ATG1a and 1b. Meanwhile, all members in the ATG18 group show a similar N-terminal structure, but lacking of a C-terminal BCAS3 domain in ATG18d, ATG18e and ATG18f, suggesting the functional divergence of different members in the same class. In a recent work, two different members in the Cysteine Proteases ATG4 group (AtAtg4a and AtAtg4b) of *A. thaliana* were approved to selectively cleavage different AtAtg8 substrates. AtAtg4a is more active when processing substrates AtAtg8a, AtAtg8c, AtAtg8d and AtAtg8i better than AtAtg4b *in vitro*, but displays similar levels with AtAtg4b when processing other AtAtg8 substrates,^33^ suggesting the function divergence of ATGs in the same group. In the present study, we found that the responses of *ATGs* in the same group to different environmental signals are distinct. Hence, the conserved and specific functions of *ATGs* in the same group need to be elucidated in the further study.

During past decades, autophagy has been shown to play roles in the adaption of plant to different stresses, including nutrient starvation, oxidative stress, drought, salt and other abiotic stresses in plants.^6^ Among them, nitrogen deficiency, starvation and darkness stress in relation to autophagy were most extensively explored, and have been used as model systems for the study of plant autophagy.^34^ It was reported that autophagy could be induced by nutrient starvation and is critical for nutrient remobilization.^35^ The relative expression levels of some *ATGs* were increased significantly under the condition of nutrient starvation. In *A. thaliana*, the transcriptional levels of *ATGs* in the ATG8 conjugation pathway were transiently unregulated in a co-ordinated manner by starvation.^36^ In *O. sativa*, the expression levels of *ATGs* were also reported to be induced by starvation stresses.^7^ However, not all *ATGs* in the ATG8 conjugation pathway were synergistically unregulated in *O. sativa.* In the present study, the responses of 30 *ATGs* in tobacco to carbon and nitrogen starvation were also investigated. Surprisingly, none of *ATGs* in the ATG8 conjugation pathway was induced by carbon and nitrogen starvation. However, we found that *ATGs* in ATG1 kinase, ATG9 recycling and PI3K complex were coordinately unregulated by both carbon and nitrogen starvation, e.g. *ATG13s* in ATG1 complex and *ATG18s* in ATG9 complex and three members of PI3K complex, which play critical roles in the initial stages of autophagy. Early study in *A. thaliana* showed that eight *ATG18* genes were differentially expressed in response to different growth conditions, and only *ATG18a* was induced by both sucrose and nitrogen starvation, which is likely required for autophagosome formation.^11^ However, six members of *ATG18* gene are commonly unregulated by both carbon and nitrogen starvation in *N. tabacum*, introducing a possibility that different members of ATG18 may cooperate in starvation induced autophagy. In addition, the responses of *ATGs* to different stresses including starvation, cold, drought, salt and heavy metals were also detected and compared with one another. We found that several *ATGs* (*ATG1a*, *ATG2*, *ATG13c*, *ATG18a*, *ATG18c* and *VPS34*) are commonly up-regulated by all abiotic stresses tested. Interestingly, all these *ATGs* also belong to ATG1 kinase, ATG9 recycling and PI3K complex, which suggested that a central role of ATG1 kinase, ATG9 recycling and PI3K complex as an effector in plant response to different stresses. In contrast, we also found that several *ATGs* are responsive only to specific stress in the present study. Interestingly, the conserved autophagy machinery could play its role in responses to different stresses, whereas several *ATGs* only response to specific stresses. This seems contradictory and remains a question with great interesting for further study. Two possible mechanisms might explain this phenomenon. The first is that the transcription of each *ATG* in different conditions may be controlled by a set of different transcriptional factors, which show different responses to various stress conditions. Another possibility is that external signals could regulate the autophagy via regulating the partners of ATGs, rather than regulate ATG expression itself. In a recent report, an ATG3-interacting protein cytosolic glyceraldehyde-3-phosphate dehydrogenases (GAPCs) has been identified, and the interaction between them was affected by ROS, which was reported to have the ability to induce autophagy.^37^ In this case, ATGs may not directly response to some stresses.

Plant hormones, as the major regulators in the different processes of plant development, have also been shown to be involved in the regulation of PCD.^38^ Two different classes of PCD, vacuolar cell death and necrosis, could be distinguished according to morphological characteristics.^39^ Necrosis usually occurs under abiotic stresses, whereas vacuolar cell death, an autophagy-like process, is common during the processes of plant development.^39^ Four major phytohormones, including SA, JA, GA_3_ and ABA, have been reported as PCD-signal regulators. SA has been approved as a well-known mediator in hypersensitive cell death,^40^ and JA usually functions in biotic stress-induced PCD.^41–43^ However, the links of plant hormones to autophagy-like vacuolar cell death remain to be elucidated. There are a few reports, indicating that GA_3_ and ABA were involved in the regulation of vacuolar cell death.^38^ In addition, the relationship between phytohormones and autophagy is also largely unknown. There are only some pioneer researches related to the relationship between phytohormones and autophagy. For example, autophagy was approved to be involved in the response to ABA.^44^ In a recent report, SA signalling pathway was also shown to be required for early senescence in *atg* mutant.^12^ In the present study, the responses of *ATGs* to ABA and SA were analysed first. The expression levels of several *ATGs* increased in seedlings after treatment by ABA and SA, respectively. However, no *ATG* gene responded to both ABA and SA synchronously, suggesting that ABA and SA regulate autophagy via different mechanisms. In addition, the responses of *ATGs* to other four phytohormones including NAA, 2, 4-D, GA_3_ and JA were comprehensively studied and compared with one another. The data implied that some *ATGs* are response to phytohormones and most of them are regulated by NAA, 2, 4-D and ABA, implying a common role of autophagy in the response to different phytohormones. The exact role of autophagy in plant response to phytohormones and the linkage between autophagy and phytohormone signals are worthy to be elucidated in the further works.

On the other hand, there are growing evidences indicating additional crucial roles of autophagy in plant development under normal conditions, including leaf starch degradation,^8^ lifespan extension,^14^ vacuolar cell death of embryo suspensor,^27^ lipid metabolism regulation, anther development^18^ and maintenance of peroxisomal quality.^15–17^ In the present study, expression profile analysis of *ATGs* in tobacco shows that the transcripts of all *ATGs* could be detected in all tissues tested, but the transcriptional levels of *ATGs* vary among different tissues especially in the processes of seed formation, which have not yet been analysed in both *O. sativa* and *A. thaliana*. The expression profile analysis results implied that autophagy has more extensive roles than we thought in plant development under normal conditions, particularly in seed development. However, clear roles of autophagy in seed development are largely unknown up to date. There are only two examples implying the role of autophagy in embryo development. In *P. abies*, autophagy has been reported to be necessary for vacuolar cell death of embryo suspensor, which is essential for normal embryogenesis. Silencing of *ATG5* or *ATG6* abrogates normal suspensor formation and consequent embryo development.^27^ Autophagy has also been reported to have a role in microspore embryogenesis.^45^ Electron microscopy observation and dansylcadaverine staining revealed that autophagy exclusively occurs in embryogenic microspores, but not in other microspore-derived structures.^45^ Hence, the exact roles of autophagy in seed formation including embryogenesis, endosperm development and seed coat development are worthy to be elucidated in the further study.

In addition, our expression profile analysis of *ATGs* in tobacco revealed that a relatively higher expression level of most *ATGs* in pollen/anther compared with that in other tissues tested, suggesting a potential role of autophagy in male gametophyte development. It was reported that *AtATG6*, one of the components of the class III phosphatidylinositol 3-kinase (PtdIns3K) complex, has been approved to be essential for pollen germination.^46–48^ Two other members of the PtdIns3K complex, *VPS15*^49^ and *VPS34*,^50^ were also shown to be important for pollen germination in *A. thaliana*. However, ATG6, VPS15 and VPS34 commonly exist in two separate complexes that regulate either autophagy or the vacuolar protein sorting pathway. In addition, the knockout mutants of other *ATG* genes in *A. thaliana* are fertile and display normal life cycle, indicating that the defects in pollen germination of *ATG6*, *VPS15* and *VPS34* are likely not attributing to the deficiency of autophagic activity in pollen. However, the contribution of autophagy to plant developmental processes may vary among different species. In *P. abies*, autophagy has been reported to be necessary for vacuolar cell death of embryo suspensor.^27^ In addition, *ATG7*-knockout mutant with defects in autophagy and sporophytic male sterility has also been identified in rice.^18^ Hence, the comparison of the roles of autophagy in different systems might be a charming work for understanding molecular mechanism underlying autophagy.

## Supplementary data

Supplementary data are available at www.dnaresearch.oxfordjournals.org.

## Funding

The work was financially supported by the National Natural Science Foundation of China (31400171 and 31270362). Funding to pay the Open Access publication charges for this article was provided by National Natural Science Foundation of China.

## Supplementary Material

Supplementary Data
