# Supplementary material for: A comprehensive, genome-wide analysis of autophagy-related genes identified in tobacco suggests a central role of autophagy in plant response to various environmental cues
Source: DNA Res. 2015 Jul 23;22(4):245–57. doi: 10.1093/dnares/dsv012 (PMC4535619; doi:10.1093/dnares/dsv012)
Supplement: Supplementary Data [file supp_22_4_245__index.html]

A comprehensive, genome-wide analysis of autophagy-related genes identified in tobacco suggests a central role of autophagy in plant response to various environmental cues — Supplementary Data 

# A comprehensive, genome-wide analysis of autophagy-related genes identified in tobacco suggests a central role of autophagy in plant response to various environmental cues

## Supplementary Data

Supplementary Data

- Supplementary Data - Doc file
