# Supplementary material for: A comprehensive, genome-wide analysis of autophagy-related genes identified in tobacco suggests a central role of autophagy in plant response to various environmental cues
Source: DNA Res. 2015 Jul 23;22(4):245–57. doi: 10.1093/dnares/dsv012 (PMC4535619; doi:10.1093/dnares/dsv012)
Supplement: Supplementary Data [file supp_dsv012_dsv012supp.doc]

**Table S1. Primers used in the present study**

| **ATG** | **For RT-PCR** | | **For RT-qPCR** | |
| --- | --- | --- | --- | --- |
|  | **Forward** | **Reverse** | **Forward** | **Reverse** |
| NtATG1a | GGGGGAAGGAGTGTGGTAGG | TGAGGATAAGAGGAGATTCTGAGG | GGGGGAAGGAGTGTGGTAGG | GGAGGCGAATGATATTAGGATGAT |
| NtATG1b | TTGTGCCGAAAATTATTGCGTTG | GGTTCCCTTCTGAGTGTCCGTGT | TGAATGAAGTTGGGAAGGCA | TGAAGGAATGATAACAAGCGGA |
| NtATG1c | CTCCTCAACCTCACTCGCCAAC | TAAAAAGGGGAACCACAAACCG | GCGTCTCTGTGTCTTCCTTTCTC | ATCCCCCGCTTCGCCTATCT |
| NtATG2 | TCCTGTCTGACCTATGTCCTTTG | GGGCAGCATTACTGTTGTCTTTA | AATCAAAAGGCTGTCTAAGGAAGT | TGTAAACGCATTGGGAGGAG |
| NtATG3 | ATCTCGTCTCCAAATGCCCTACT | CAACTTCTGGTTCAACTCCCCGT | ACAAGCCCTCGCACCGTTTCT | GCCAGCCATCATTTTCCTCATTA |
| NtATG4 | GTGATATTTGGCTTTTAGGTGTTTG | GTAAGTCGAGGCTCCAGGTCTG | AAGAGAGAAGAAACGGGGAATG | CCAACGGAACTAAGAAAAGAACAG |
| NtATG5 | ACTCTCCCTTCTCCTCCCCCTG | TTGTATTGAAAAATCATCCACCAC | ATGGGAGGTAAAGGAGCAGGAGG | AGGGGGAGGAGAAGGGAGAGTAG |
| NtATG6 | TTATCCTCGGATCATGGACACC | CAAACCAGTAGAGCACCCACTTC | GGTTTCACTCCACCATCACTGT | TTTCGCTCTTCTTCTTCTATCTTCA |
| NtATG8a | TCCTTCAAATTGGAACACCCAC | TCCTCATAAATCGCAGACATCA | CCTGCTGATCTGACTGTGGG | CTGTCGGAGGAAGGATATTTTTC |
| NtATG8b | AGTTGGGCAATTTGTCTATGTC | CGAATGTGTTTTCTCCACTGTAA | CAGTTGGGCAATTTGTCTATGTC | TTCAGGTCCCCGAATGTGTT |
| NtATG8c | TTAGGGAAAAGTACCCCGATAGGATTC | GTTGGCGGTAGGACATTGTCAAC | TATTCCCAACATTGACAAGAAAAAG | TGACGTAGACAAACTGCCCCA |
| NtATG8d | CATCCGAGAGAAGTATCCCGATC | GAGGCAAAGTGTTGTTGACAAAG | CATCCGAGAGAAGTATCCCGA | CAGACAACAGAGCAGCCGTG |
| NtATG8e | ATCCTTTGGAGAGGAGGCAGGCA | GCAGACATCAGAGCAGCAGTTGG | TTTGGAGAGGAGGCAGGCA | CAGACATCAGAGCAGCAGTTGG |
| NtATG9 | TTCTCCTATCTCCGTTTCTCGT | CTCATCTGTTATTGCAGCCCTG | CTTGACCCACAGGGAGCGA | CAGTGAAGTCCACAATGAAATGC |
| NtATG10 | CAAAAGCACAGCCAGGAAAGAC | TTAAGACCAAAGATCGGACACAC | CTACAGTTCCTCTTTCAGGGTTCC | TCTTTCCAGGTCTTCTTTTGCC |
| NtATG13a | TTATGGTCGTAGTGGAGAGGTG | TCATTGAATATAGGGAGCGGAG | GATGTCTTTGATTTTTCCTGTCCTT | CCCTCCACTTCAGTCTTTACGC |
| NtATG13b | GTGGGGAAGTGAAAAAGAGTG | TGAATAAAGCGAGCGGAGTAG | TTCTCTCCCACCTATGTCACCTA | AAACCGTCCTGAATCGTCTCTA |
| NtATG13c | TTCATTTAGTGCTCCATTCTCC | CTGGTACTCATCAAAGCTCGTC | ATACACCACAAAGCGACGAGC | GGGAGACATTGGAGGGAGAGA |
| NtATG18a | AGAGAGGCACAACTCGGCAAAT | GCAGGAAACCCCGTGACAACAG | TGGTGGTAGTAACAGTGGGGAG | CCAAACAACAGTAGGCAGAAAAG |
| NtATG18b | GTCCATAGCTTCAGCTTTTCGC | TCAACCTCAGCCTCTCCACCAC | GCTAGATTTGATACCAACTATTCCTGC | CATTTCCCTTTGTTATTCCTTCA |
| NtATG18c | ACCCATACTTCTACCTATTCTTTCG | ATGTCTGTTACTTCACGGCCA | CGGTGTTATCTCCTCAGGTTTTC | CATTGGTATTTGTCCCATTCTTC |
| NtATG18d | CCAGGACTAACGCCCAATACC | TTTGTCAACCCCTCTACGCAC | ATCAGGATTACAGTTGCTTTGCT | TTATTAGGTGGGTATTGGGCG |
| NtATG18e | AACACAAACAACAGCTTCAGCC | TTATCTCCACTTCCAACAAAGACTAG | AAAACCCCACAGGTCCCAG | AGATAGTCACCACCACATTCACG |
| NtATG18f | TATAATGGCACATGACTCGAGGA | AATGAAGGAGAGATGAGACACAGC | GAAGGGTCAGGTGAGAGTGGA | TGAAGAAAAGGCAAGGCTATAGA |
| NtATG20 | CATTTTTATTCCTCCACTTCCAG | TGAGCATTTCATACTCACGCTC | CCGAGGCTGAGCGTGAGTA | AAGAAGACTTCGCCATCCATC |
| NtVTI12a | GATTCGCAAGATGGACCTAGAAGC | ACAGGATAGCCAGGACCAAGACAG | CAAGAAGGATGAGCAGGAATAAA | GAACTAACAGGATAGCCAGGACC |
| NtVTI12b | GCCGAAAAAGCAACAGACATCC | GCTCCAATTACAGAGCCCATAATC | AGACCTTGGTGTCTCAATCCTTG | CACTTGTTTCGGCTAATCCTTC |
| NtVPS15 | TTGGACAACGAAGTGTGGAAGAAT | GGGAAGTAAATGGCAATGAGGAAT | CCTCGTGGGGTTTTGGTTG | ACTGTGGTCTGCTGAGATGGAT |
| NtVPS34 | CATTGTATATAGATGGTGCTCCCT | AAGCTTTAGTTGTTTGTGCTCAGA | CTACAGAAAGAAAGTCAATACAAAGAA | CGCTAACGGCATAGGCACG |
| NtTOR | GGGCCTCGGCTGTGTCCCTTATT | TGCATGTCCGTCCCAACCTCGTA | CTGTCATTCCATTGTCCCCTAA | GCGTATTCAAACACCTCCACC |


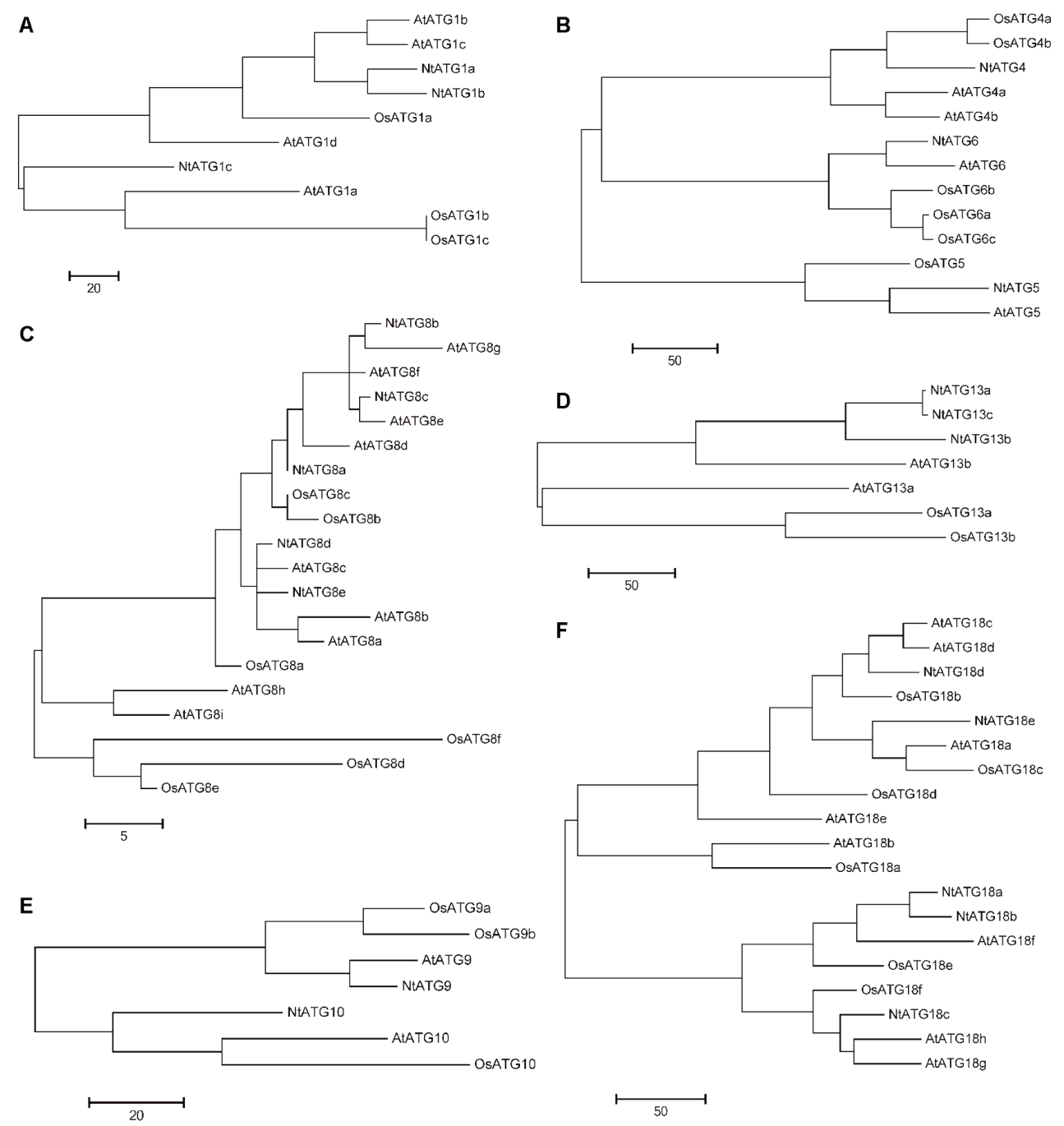


**Figure S1. Phylogenetic relationship of different ATGs from *O. sativa*, *N. tabacum* and *A. thaliana***

The tree was calculated with MEGA 5.1 software using maximum parsimony method.
